# Radiomics‐based model for accurately distinguishing between severe acute respiratory syndrome associated coronavirus 2 (SARS‐CoV‐2) and influenza A infected pneumonia

**DOI:** 10.1002/mco2.14

**Published:** 2020-08-13

**Authors:** Qi‐Qiang Zeng, Kenneth I. Zheng, Jun Chen, Zheng‐Hao Jiang, Tian Tian, Xiao‐Bo Wang, Hong‐Lei Ma, Ke‐Hua Pan, Yun‐Jun Yang, Yong‐Ping Chen, Ming‐Hua Zheng

**Affiliations:** ^1^ Clinical Research Center The Second Affiliated Hospital of Wenzhou Medical University Wenzhou China; ^2^ NAFLD Research Center, Department of Hepatology The First Affiliated Hospital of Wenzhou Medical University Wenzhou China; ^3^ School of the First Clinical Medical Sciences Wenzhou Medical University Wenzhou China; ^4^ Department of Critical Care Medicine Wenzhou Central Hospital Wenzhou China; ^5^ Department of Radiology The First Affiliated Hospital of Wenzhou Medical University Wenzhou China; ^6^ Institute of Hepatology Wenzhou Medical University Wenzhou China; ^7^ Key Laboratory of Diagnosis and Treatment for The Development of Chronic Liver Disease in Zhejiang Province Wenzhou China

**Keywords:** COVID‐19, diagnosis, SARS‐CoV‐2

## Abstract

Clinicians have been faced with the challenge of differentiating between severe acute respiratory syndrome associated coronavirus 2 (SARS‐CoV‐2) infected pneumonia (NCP) and influenza A infected pneumonia (IAP), a seasonal disease that coincided with the outbreak. We aim to develop a machine‐learning algorithm based on radiomics to distinguish NCP from IAP by texture analysis based on computed tomography (CT) imaging. Forty‐one NCP and 37 IAP patients admitted from January to February 6, 2019 admitted to two hospitals in Wenzhou, China. All patients had undergone chest CT examination and blood routine tests prior to receiving medical treatment. NCP was diagnosed by real‐time RT‐PCR assays. Eight of 56 radiomic features extracted by LIFEx were selected by least absolute shrinkage and selection operator regression to develop a radiomics score and subsequently constructed into a nomogram to predict NCP with area under the operating characteristics curve of 0.87 (95% confidence interval: 0.77‐0.93). The nomogram also showed excellent calibration with Hosmer‐Lemeshow test yielding a nonsignificant statistic (*P* = .904). The novel nomogram may efficiently distinguish between NCP and IAP patients. The nomogram may be incorporated to existing diagnostic algorithm to effectively stratify suspected patients for SARS‐CoV‐2 pneumonia.

## Graphical abstract

Clinicians have been faced with the challenge of differentiating between severe acute respiratory syndrome‐associated coronavirus 2 (SARS‐CoV‐2) infected pneumonia (NCP) and influenza A infected pneumonia (IAP). We developed a machine‐learning algorithm based on radiomics to distinguish NCP fromIAP by texture analysis based on computed tomography (CT) imaging. Eight of 56 radiomic features extracted by LIFEx were selected to develop a radiomics score and subsequently constructed into a nomogram to predict NCP. The novel nomogram may efficiently distinguish between NCP and IAP patients.

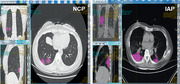

**Abbreviations**
ASA: American Society of Anesthesiologists
AUC: area under the operating characteristics curve
CT: computed tomography
IAP: influenza A virus infected pneumonia
LASSO: least absolute shrinkage and selection operator
NGLDM: neighborhood gray‐level dependence matrix
ROI: region of interest
SARS‐CoV‐2: severe acute respiratory syndrome‐associated coronavirus 2
SVM: support vector machine

## 1 INTRODUCTION

Recently, the spread of coronavirus disease 2019 (COVID‐19), caused by severe acute respiratory syndrome associated coronavirus 2 (SARS‐CoV‐2), has become a global pandemic and public health problem.^1^, ^2^, ^3^ As of March 12, 2020, a total of more than 180 000 cumulative confirmed cases and 4292 deaths for COVID‐19 had been reported globally.^4^ Although unprecedented efforts had been concentrated to identify and isolate individuals with risk of SARS‐CoV‐2 infection, clinicians are facing tremendous difficulties in efficiently and quickly diagnosing these patients due to massive volume of suspected cases.

Accurately diagnosing pneumonia patients is paramount in that those without SARS‐CoV‐2 infection may be exposed to risk of cross‐infection during inpatient and group isolation as human‐to‐human has been reported to be the most prominent route of transmission.^2^ The current major challenge is differentiating between patients with NCP and those with influenza A infected pneumonia (IAP), a seasonal disease that coincided with SARS‐CoV‐2 outbreak.^5^ This is clinically important because there is little evidence that early clinical signs and symptoms demonstrated by NCP patients are any different from that of their IAP counterparts. Computed tomography (CT) is often used to evaluate the pulmonary condition of those suspected SARS‐CoV‐2 infection. However, clinicians may also find challenges in distinguishing radiographic features of NCP patients from pneumonia of other viral etiologies.^6^, ^7^

We aim to develop an effective diagnostic tool to overcome the above‐mentioned challenge using machine learning methods based on CT radiomics. We hope our findings may aid diagnosticians currently serving first‐line against SARS‐CoV‐2 outbreak.

## 2 METHODS

### 2.1 Study design and participants

Forty‐five SARS‐CoV‐2 positive and 132 influenza A virus positive patients admitted to the First Affiliated Hospital of Wenzhou Medical University and Wenzhou Central Hospital from January 1 to February 6, 2020, were included in this study. Patient's demographical, clinical, laboratory, and radiological data were recorded and reviewed by trained physicians upon admission and prior to receiving any medical treatment. Retrieval of above‐mentioned data for this study was performed retrospectively from electronic medical records. Patients with SARS‐CoV‐2 infection were diagnosed by confirmatory test according to recommendations set forth by the World Health Organization interim guidance.^8^ Influenza A viral infection was confirmed by measuring nucleic acid by fluorogenic quantitative PCR. Pneumonia was diagnosed by radiological evidence of lung inflammatory lesions with or without pleural effusion. Patients were excluded if they met any of the following criteria: (a) history of American Society of Anesthesiologists (ASA) score of more than 2; (b) history of existing respiratory disease prior to outbreak of SARS‐CoV‐2; (c) pneumonia of etiology other than SARS‐CoV‐2 or influenza A virus by measuring nucleic acid by fluorogenic quantitative PCR of serum samples and/or oropharyngeal swab samples in conjunction to radiographic evidence and clinically established diagnosis; or (d) absent of obvious pulmonary lesions on radiographic imaging.

The First Affiliated Hospital of Wenzhou Medical University and Wenzhou Central Hospital are situated in Wenzhou, China, and the former is the largest staffed teaching tertiary hospital designated for the treatments of NCP by local government.^9^ This study was approved by the local ethics review board by First Affiliated Hospital of Wenzhou Medical University and Wenzhou Central Hospital, waiving patient written informed consent for deidentified data.

### 2.2 Real‐time reverse transcription PCR assay for SARS‐CoV‐2

In brief, throat swab samples were collected from patients and preserved in appropriate solution prior to transport to central laboratory. Total RNA was extracted and subsequently tested by real‐time reverse transcription PCR assay (Shanghai ZJ Bio‐Tech Co., Ltd.), according to diagnostic guideline set forth by the Chinese National Institute for Viral Disease Control and Prevention (http://ivdc.chinacdc.cn/kyjz/202001/t20200121_211337.html).

### 2.3 CT acquisition

**FIGURE 1:**
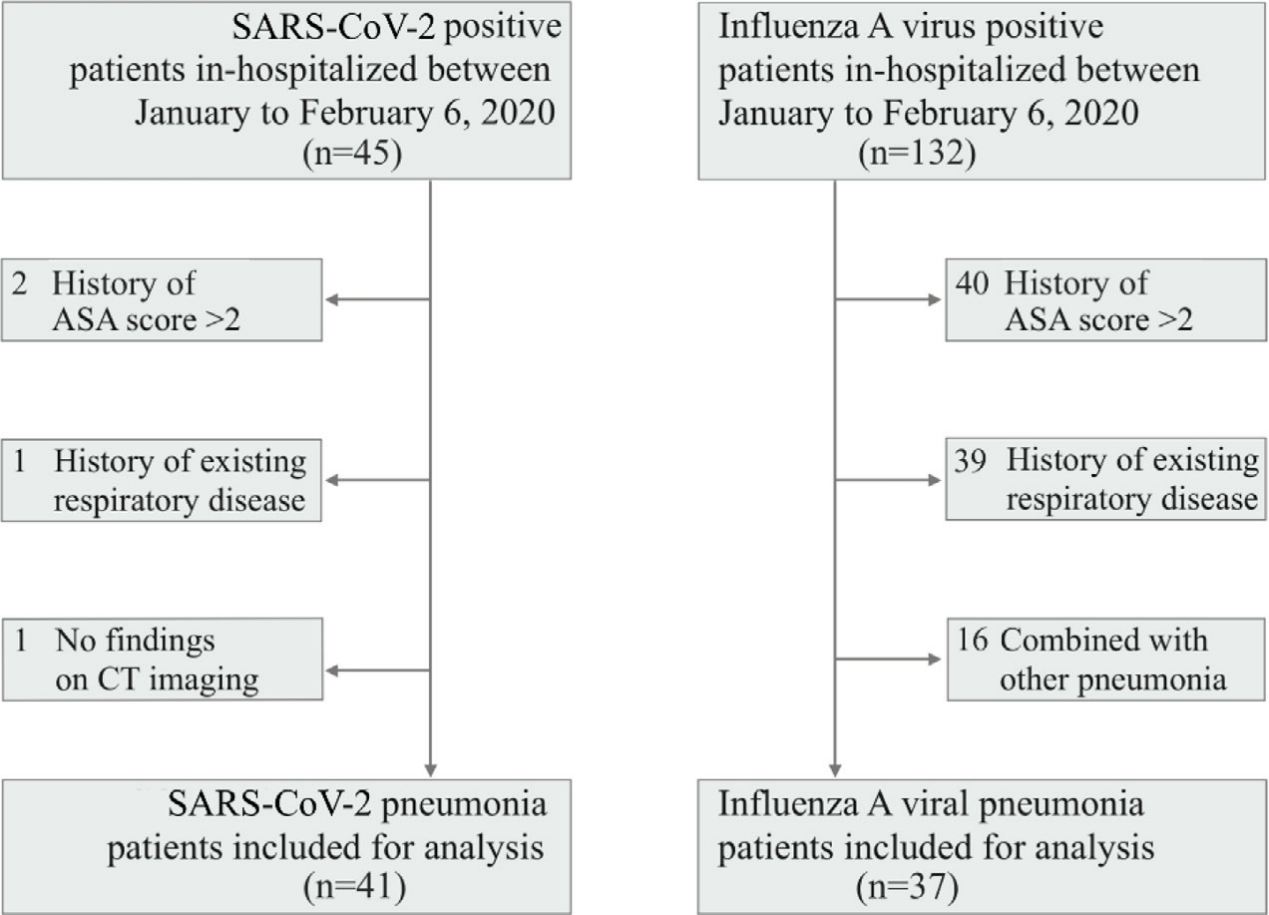
Enrollment flow diagram of the study. Abbreviation: ASA score, American society of anesthesiologists score

In brief, all patients underwent chest CT scans using 64‐slice CT scanner (Light‐speed; GE Healthcare, Chicago, IL) with a collimation coverage of 40 cm and reconstruction thickness of 1.25 mm and 5 mm, while holding breath and lying in a supine position. Parameters used for CT varied with patient size and were, on average, 120 kV with mAs 100‐250. Digital CT imaging was exported from Picture Archiving and Communication System without any processing or standardization. Prior to radiomic feature analysis, imaging segmentation on above‐mentioned exports were performed by an expert radiologist and validated by another senior radiologist with at least 10 years of experience. Region‐of‐interest (ROI) selection was conducted covering all detectable inflammatory lesions of the lungs prior to texture analysis. The machine learning procedures and workflow are illustrated in Figure 1.

### 2.4 Texture feature extraction and radiomic signature selection

Based on segmented ROI, texture features were extracted by LIFEx (version 5.0) using open script (https://www.lifexsoft.org/index.php/resources/19-texture/radiomic-features). LIFEx is an open source and multiplatform freeware developed as a tool to mine radiomic texture data from established medical imaging.^10^ The texture analysis by LIFEx consisted of a two‐order evaluation resulting in 56 three‐dimensional radiomic features. The first‐order involved measuring shape histogram based matrix and histogram‐based matrix. The second order assessed gray‐level co‐occurrence matrix gray‐level zone length matrix, neighborhood gray‐level dependence matrix (NGLDM), and gray‐level run length matrix. The overall signature analysis enables differentiation of pulmonary inflammatory lesion attributed to the two distinct etiological pneumonias (Supplementary figure 1). For simpler procedural replication and reproducibility, detailed extraction techniques and typesetting may be found in the Texture User Guide of LIFEx (www.lifexsoft.org).

Least absolute shrinkage and selection operator (LASSO) with 10‐fold cross‐validation was employed for the radiomic signature/feature selection. The utility of LASSO regression model begins with identification of optimal penalization coefficient lambda (λ) among multitude of radiomic features extracted by LIFEx. By adjusting λ, LASSO is able to differentiate signatures that do not associate with NCP by shrinking their coefficients to zero. Thus, signatures with nonzero coefficient are subsequently selected for establishment of a radiomics score.

**Key points**

- Chest computed tomography can be used as a quick tool to screen patients for pneumonia.
- Influenza A viral pneumonia have similar clinical presentation as SARS‐CoV‐2 pneumonia.
- Radiomics‐based model of the chest computed tomography can be utilized to effectively differentiate between SARS‐CoV‐2‐infected pneumonia. This may help stratify pneumonia patients for COVID‐19, especially when nucleic acid testing kits (confirmatory test for SARS‐CoV‐2) is unavailable.

### 2.5 Construction and validation of the nomogram

Predictive features selected by the LASSO regression were subject to logistic regression for development of radiomic score. Further evaluation was performed by waterfall plot for assessing the linear association between radiomics score and observed disease outcome. Each diseased patient was plotted against, respectively, associated radiomics score for better visualization of their associative qualities. The components deemed valuable to outcome prediction were used for the final construction of nomogram. The performance of the nomogram in discriminating NCP and IAP were evaluated by area under the operating characteristics curve (AUC) and subjected to calibration.

### 2.6 Support vector machines classification of SARS‐CoV‐2 and IAP

The support vector machine (SVM) is a state‐of‐the‐art algorithm used to classify disease outcome by sorting differentiative coordinate mapped by massive amount of radiomics features. The SVM is capable of plotting n‐dimensional space where n represents the number of radiomic features available for assessment. In this study, we performed classification by running SVM algorithm (C‐classification and radial kernel on Python 3.7.5) based on radiomic features resulted from LASSO regression and logistic regression. A visualization plot was subsequently drawn to compare the predicted outcome versus observed outcome of each patient in order to illustrate the probability of NCP. The capacity of prediction models in separating patients with NCP and IAP was evaluated by the discrimination demonstrated by the SVM plot.

### 2.7 Statistical analysis

Categorical variables were described as frequency rates and percentages, and continuous variables were described using median and interquartile range (IQR) values. Difference between categorical variables was examined with the chi‐squared test or Fisher's exact test as appropriate. A *P*‐value < .05 was considered statistically significant. Data management and analysis were performed using R software (R version 3.5.2, R Foundation for Statistical Computing, Vienna, Austria) and Python 3.7.5.

## 3 RESULTS

### 3.1 Patient characteristics

Four of the 45 SARS‐CoV‐2 patients were excluded (two for history of ASA score > 2, one for history of existing respiratory disease, and one for absent of obvious findings on CT imaging). Of the 132 influenza A viral infected patients, 95 were excluded (40 for history of ASA score > 2; 39 for history of existing respiratory disease; and 16 for pneumonia of other etiology) (Figure 3). As a result, 41 NCP and 37 IAP patients were included for the final analysis.

**FIGURE 2:**
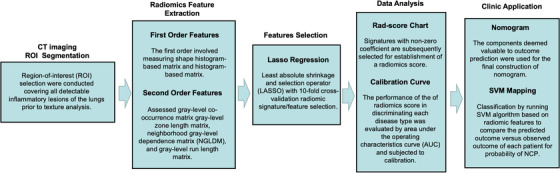
Radiomics‐based machine learning workflow, including computed tomography (CT) images acquisition and region‐of‐interest (ROI) segmentation of inflammatory lesions; radiomic feature extraction by LIFEx; features selection by least absolute shrinkage and selection operator (LASSO) with 10‐fold cross‐validation; radiomics prediction score and calibration; and nomogram development for a more clinician‐friendly application, and support vector machine (SVM) were used to distinguish these two kinds of diseases effectively

Table 1 summarizes the main clinical and biochemical characteristics of pneumonia patients stratified by etiology. Overall, NCP and IAP patients share similar clinical and laboratory variables. The median age for NPC and IAP patients were 46 (IQR, 39–50) and 55 (IQR, 45–67) years, respectively, *P* < .01. The most common symptoms found in included pneumonia patients, irrespective of etiology, were fever, fatigue, cough, myalgia, and dyspnea; however, no significant difference was observed between NCP and IAP patients except in proportions of patients showing symptoms of cough (22 [53.7%] vs 31 [83.8%], *P* < .01]. Of the lesser common symptoms, higher proportion of dyspneic patients was observed in the IAP group. By comparing the laboratory parameters between NCP and IAP patients, difference in serum levels of white blood cell count and aspartate aminotransferase was observed. However, no relationship can be observed between disease condition and above‐mentioned differences in biochemical profile.

**TABLE 1:** Demographics and baseline characteristics of patients infected with SARS‐CoV‐2 or influenza A virus

	SARS‐CoV‐2 (n = 41)	Influenza A (n = 37)	*P*‐value
Characteristics			
Age, years	46 (39‐50)	55 (45‐67)	<.01
Sex			.40
Men	15 (36.6%)	17 (45.9%)	
Women	26 (63.4%)	20 (54.1%)	
Signs and symptoms			
Fever	32 (78.0%)	28 (75.7%)	.80
Highest temperature, °C			.70
<37.3	9 (22.0%)	9 (24.3%)	
37.3‐38.0	15 (36.6%)	11 (29.7%)	
38.1‐39.0	11 (26.8%)	8 (21.6%)	
>39.0	6 (14.6%)	9 (24.3%)	
Cough	22 (53.7%)	31 (83.8%)	<.01
Myalgia or fatigue	11 (26.8%)	8 (21.6%)	.61
Headache	2 (4.9%)	2 (5.4%)	.92
Hemoptysis	1 (2.4%)	3 (8.1%)	.34
Diarrhea	2 (4.9%)	0 (0.0%)	.50
Dyspnea	1 (2.5%)	15 (40.5%)	<.01
Respiratory rate > 24 breaths per min	1 (2.5%)	4 (10.8%)	.19
Laboratory data			
White blood cell count, ×10^9^/L			.02
<4	13 (32.50%)	10 (27.03%)	
4‐10	26 (65.00%)	18 (48.65%)	
>10	1 (2.50%)	9 (24.32%)	
Lymphocyte count, ×10^9^/L			
<1.0	5 (12.50%)	1 (2.70%)	.20
≥1.0	35 (87.50%)	36 (97.30%)	
Aspartate aminotransferase, U/L			.05
≤40	31 (81.58%)	22 (59.46%)	
>40	7 (18.42%)	15 (40.54%)	
Total bilirubin, mmol/L	10.2 (7.1‐15.0)	8.0 (6.0‐12.0)	.07
Lactate dehydrogenase, U/L			.40
≤245	20 (62.50%)	10 (47.62%)	
>245	12 (37.50%)	11 (52.38%)	

*Note*. Continuous variables are presented as median (IQR), n (%); categorical variables are presented as number (%). *P* values tested by one‐way ANOVA for normally distributed variables, Kruskal‐Wallis rank test for not normally distributed continuous variables, and Fisher's exact test for categorical variables, respectively.

### 3.2 Radiomic feature construction

Based on CT imaging derived ROI, 56 radiomic features were extracted and compared (Supplementary figure 1). The analysis by least absolute shrinkage and selection operator (LASSO) with 10‐fold cross‐validation of these 56 radiomic features yield eight distinct features that were most associative to NCP (Figure 2). The eight distinct features were subsequently used to build a radiomics score producing the equation as follows: −0.84557 + 0.00219*CONVENTIONAL_HUMIN + 0.00145*CONVENTIONAL_HUMAX −0.00623*CONVENTIONAL_HUQ1 −0.32549*HISTO_EXCESSKURTOSIS −106.08669*GLRLM_LRLGE + 455.66502*NGLDM_COARSENESS + 0.60757*NGLDM_BUSYNESS −0.00002*GLZLM_ZLNU. To better visualize the associative quality of the radiomics score and NCP, a waterfall plot was drawn. As illustrated in Figure 4A, the observation can be made that only minor disagreements exist when each patient was plotted against respective radiomics score. Therefore, it is reasonable to infer that radiomics score possesses satisfactory predictive capability to differentiate NCP and IAP patients.

**FIGURE 3:**
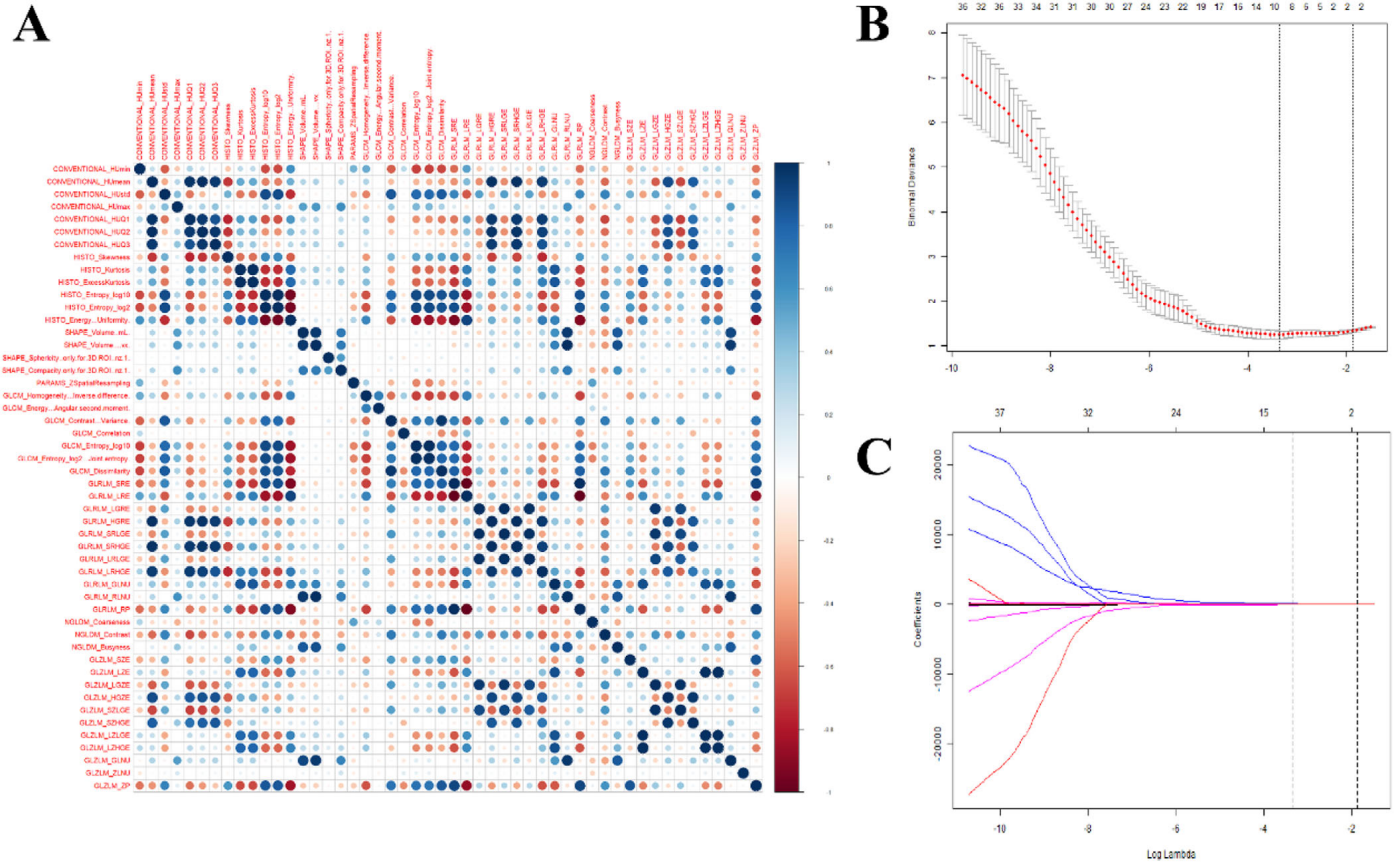
Radiomic feature selection from signature heatmap using the least absolute shrinkage and selection operator (LASSO) logistic regression model. (A) The heat map of relationship among texture analysis parameters. (B) Identification of the optimal penalization coefficient lambda (λ) in the LASSO model used 10‐fold cross‐validation and the minimum criterion. (C) Lasso coefficient profiles of the 56 radiomic features. The dotted vertical line was plotted at the value selected using 10‐fold cross‐validation in (A), for which the optimal λ resulted in 10 non‐zero coefficients

**FIGURE 4:**
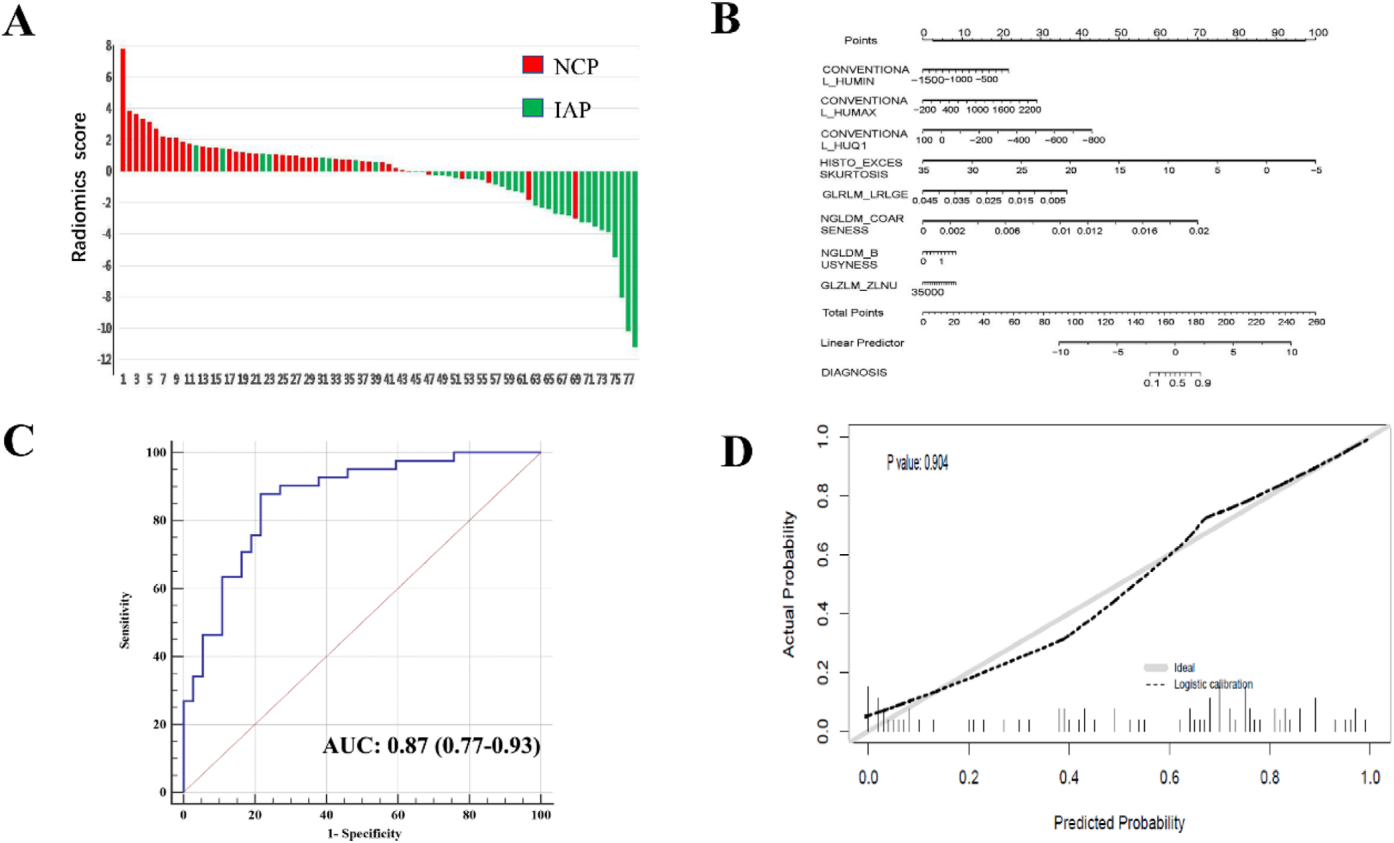
(A) Waterfall plot of radiomics score for each patient. Default is set to NCP (red bar) above the baseline and IAP (green bar) below the baseline. This plot assesses the association between radiomic score and disease type in which disagreement of color coding indicates misclassification by the radiomics score. (B) Nomogram for differentiating NCP and IAP. For each patient, the value of eight variables are represented as points by projecting them onto the upper‐most line (Points). Summing the eight variables and projecting the total points value downward onto the bottom‐most line (DIAGNOSIS) can determine disease type. Value approaching 0 on the DIAGNOSIS line indicates higher probability of IAP while approaching 1 indicates higher probability of NCP. Linear predictor is the nomogram visualization of NCP prediction by radiomics score. (C) Receiver operating characteristic curve for predicting NCP by nomogram; AUC is expressed as n (95% confidence interval). (D) Calibration curve of the nomogram. Calibration curves depict the calibration of model in terms of the agreement between the predicted risks of novel coronavirus and observed outcomes. The *y*‐axis represents the actual novel coronavirus rate. The *x*‐axis represents the predicted novel coronavirus risk. The diagonal solid line represents a perfect prediction by an ideal model. The dotted line represents the performance of the nomogram, of which a closer fit to the diagonal solid line represents a better prediction. The Hosmer‐Lemeshow test yielded a nonsignificant statistic (*P* = 0.904), which suggested that there was no departure from perfect fit. Abbreviations: NCP, SARS‐CoV‐2 infected pneumonia; IAP, influenza A infected pneumonia; AUC, area under operating characteristics curve

### 3.3 Nomogram development

A clinician user‐friendly nomogram was developed to visually quantify the individualized probability of a patient for having NCP (Figure 4B). The nomogram incorporated eight predictive components of the radiomics score in order to optimize the diagnostic utility in differentiating disease outcome (NPC vs IAP). Based on summation of “Total Points” on the nomogram, its corresponding value on the line “DIAGNOSIS” may produce a quantifiable risk assessment for pneumonia etiology; as the value approaches 1, it is more likely to be NCP.

### 3.4 Diagnostic performance of models

**FIGURE 5:**
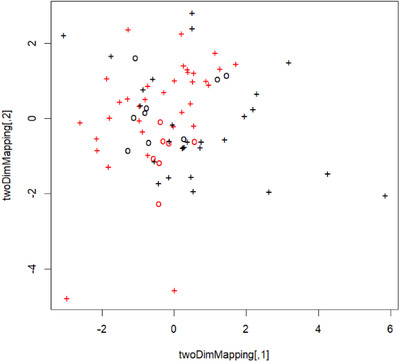
Support vector machine visualization plot. Black cross indicates patients with IAP; red cross indicates patients with NCP; circle indicates misclassification or incorrect prediction; the *X*‐ and *Y*‐axis denote mapping variables for two‐dimensional presentation of the multidimensional hyper‐plane

As illustrated in Figure 4C, the nomogram achieved excellent performance in predicting NCP with area under the characteristic curve (AUC) of 0.87 (95% confidence interval: 0.77‐0.93). The diagnostic ability of nomogram was evaluated alongside calibration curve (Figure 4D). Hosmer‐Lemeshow test yielded a nonsignificant statistic (*P* = .904) for the nomogram, which suggested excellent discrimination for both NCP and IAP. Figure 5 represents a two‐dimensional projection by VSM. The plotted visualization depicts the comparison of predicted outcome versus observed outcome of each patient, and plotted illustration indicated a satisfactory performance for the diagnostic model.

## 4 DISCUSSION

To the best of our knowledge, this is the first observational study to investigate the radiomic signatures on CT imaging of NCP patients. This is also the first study to propose a diagnostic model to distinguish between NCP and IAP patients. We found eight of 56 signatures, by analyzing radiomics features on CT imaging, are independent diagnosticator of NCP and subsequently developed a radiomics score that may accurately classify pneumonias of the two examined viral etiologies. To ensure realistic clinical application of our diagnostic tool, we established a user‐friendly nomogram to aid clinicians in stratifying patients for NCP. Our data show that the nomogram has excellent diagnostic accuracy for differentiating between NCP and IAP patients, and by using this tool clinicians may make better decision on choosing the next step of management.

While the source of the SARS‐CoV‐2 is actively under investigation, drastic health security measures have been implemented across China in response to containing all possible contagion. In areas of higher outbreak activities, such as in the case of Wenzhou, China, local authorities moved quickly to close its borders and imposed a conditional city‐wide curfew to reduce public human gatherings.^11^ As a result, the city ushered a steady decrease in reported incidence of NCP patients from January 31 to February 10, 2020 (55 vs 16 new daily cases reported on January 31 and February 10, 2020, respectively).^12^ However, worrisome local reports identified new cases of NCP in those who were previously tested negative for SARS‐CoV‐2. Although multiple factors may contribute to this finding, including false negative real‐time RT‐PCR results and posthospital infection, it is possible that some of the new cases of NCP were the result of hospital‐related cross‐infection during initial visitation. Recent epidemiological report by Wang et al showed that among 138 confirmed NCP patients, prevalence of presumed in‐hospital infection was observed to be as high as 41.3%.^12^ Thus, based on these preliminary findings, we can make the following inference: (1) real‐time RT‐PCR testing does not completely rule out SARS‐CoV‐2 infection and complementary diagnostic strategies may be required; and (2) accurately differentiating pneumonia by SARS‐CoV‐2 from other etiologies and separately hospitalize these patients may reduce the likelihood of in‐hospital cross‐infection. That said, pneumonia patients entering in‐hospital observation without effective risk stratification for NCP may place those without SARS‐CoV‐2 infection at greater risk for cross‐infection.

In China, there were approximately 88 100 influenza‐associated excess respiratory deaths per year, and the most prevalent influenza A virus, with strong winter seasonality, has an estimated mortality rate of up to 3.9 (95% CI: 3.6‐4.2) per 100 000 person‐seasons.^13^ Common radiographic features of IAP patients on chest CT include ground‐glass opacities and consolidation, which are not outstandingly different from that of NCP patients to the naked eye, as a recent report demonstrated.^6^, ^7^ As a consequence, the seasonal flu pneumonia combined with the outbreak of SARS‐CoV‐2 have produced a tremendous challenge for diagnosticians across China. This is because there is little evidence that early clinical signs and symptoms demonstrated by IAP patients are any different from that of their NCP counterparts. More pertinently, IAP patients that are isolated for observation could be exposed to SARS‐CoV‐2 cross‐infection. Although it is currently unclear the clinical outcome for patients with combined NCP and IAP, it is reasonable to speculate that IAP patients (often at subpar immune state) with superimposed SARS‐CoV‐2 infection are at increased risk for mortality. That said, accurately diagnosing and differentiating between NCP and IAP patients is paramount.

To solve this conundrum, we have developed a diagnostic tool using machine learning methods based on radiomics that may effectively and accurately distinguish between NCP and IAP patients. Although real‐time RT‐PCR testing lacks the ability to diagnose pneumonia by design, it possesses good sensitivity and remains currently the gold standard for confirming SARS‐CoV‐2 infection. That said, our proposed diagnostic tool may be used as a screening assessment for patients suspected of NCP prior to real‐time RT‐PCR testing or used as a complementary examination for better evaluation of patient's condition. By accurately diagnosing NCP, number of suspected patients without SARS‐CoV‐2 may be reduced and consequently reduce the risk of hospital‐related cross infection.

There are several limitations to our results. The scarcity of available NCP (n = 41) and IAP (n = 37) patients for analysis and the lack of external validation may limit the generalizability of our diagnostic model; future validation studies are certainly needed. By design, our proposed diagnostic tool is developed for differentiating between NCP and IAP; therefore, precaution is needed prior to evaluating patients with viral pneumonia of etiologies other than above‐mentioned pathogens. Our study, while limited mostly by small sample size, has a few notable strengths. First, radiomics may provide more detailed and specific information, patterns, and signatures on radiography that are often not apparent to the naked eye, and combining it with machine learning methods can provide superior modeling tools for improved accuracy. Second, to promote transparency, we included detailed and replicable procedures (open script) for texture analysis in order to conduce higher reproducibility and quicker implementation. Lastly, this study is clinically important because our proposed diagnostic tool may enable clinicians to quantitatively differentiate SARS‐CoV‐2 and IAP on CT imagining, which was not realizable until now.

In conclusion, the novel nomogram, developed based on radiomics, may accurately distinguish NCP and IAP patients as demonstrated in this study. This tool is easily reproducible, therefore, is conducive to widespread clinical implementation. Potential application includes screening or diagnosing suspected patients in an algorithm combining real‐time RT‐PCR to effectively stratify suspected patients for COVID‐19.

## AUTHOR CONTRIBUTIONS

Concept and design: Zeng, Chen, and Zheng. Acquisition and interpretation of data: Zeng, Chen, Jiang, Tian, Wang, Ma, Pan, and Yang. Drafting of the manuscript: Zheng. Statistical analysis: Zeng, Ma, and Bai. Critical revision of the manuscript for important intellectual content: Zeng, Zheng, and Zheng. All authors reviewed the manuscript and approved the final version.

## GUARANTOR

The scientific guarantor of this publication is Prof. M.H. Zheng.

## CONFLICT OF INTEREST

The authors declare no conflict of interest.

## Supporting information

Supplementary figure 1
