# Supplementary material for: Radiomics‐based model for accurately distinguishing between severe acute respiratory syndrome associated coronavirus 2 (SARS‐CoV‐2) and influenza A infected pneumonia
Source: MedComm (2020). 2020 Aug 13;1(2):240–8. doi: 10.1002/mco2.14 (PMC7436469; doi:10.1002/mco2.14)
Supplement: Supplementary file 1 — Supplementary figure 1 [file MCO2-1-240-s001.pptx]

## Slide 1
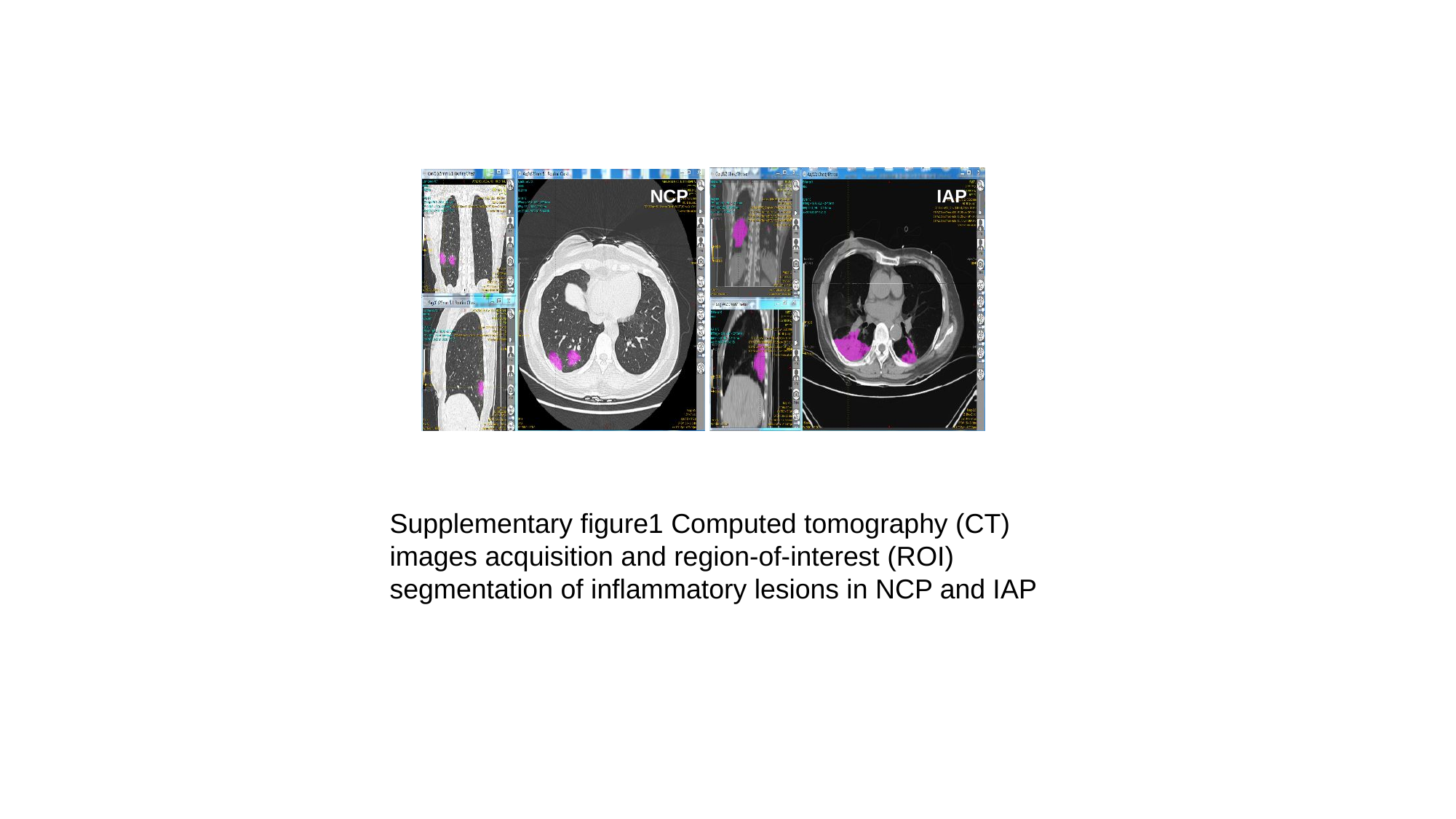

NCP IAP
Supplementary figure1 Computed tomography (CT) images acquisition and region-of-interest (ROI) segmentation of inflammatory lesions in NCP and IAP
